# A high throughput method for quantifying number and size distribution of *Arabidopsis* seeds using large particle flow cytometry

**DOI:** 10.1186/s13007-020-00572-x

**Published:** 2020-03-02

**Authors:** Alejandro Morales, J. Teapal, J. M. H. Ammerlaan, X. Yin, J. B. Evers, N. P. R. Anten, R. Sasidharan, M. van Zanten

**Affiliations:** 1 Centre for Crop Systems Analysis, Plant Sciences Group, Wageningen University & Research, Wageningen, The Netherlands; 2 Plant Ecophysiology, Institute of Environmental Biology, Utrecht University, Utrecht, The Netherlands; 3 Molecular Plant Physiology, Institute of Environmental Biology, Utrecht University, Utrecht, The Netherlands; 4 Developmental Biology, Institute of Biodynamics and Biocomplexity, Utrecht University, Utrecht, The Netherlands

**Keywords:** Machine learning, BioSorter, R package, Phenotyping, *SeedSorter*, Seed number, Seed size, *Arabidopsis thaliana*

## Abstract

**Background:**

Seed size and number are important plant traits from an ecological and horticultural/agronomic perspective. However, in small-seeded species such as *Arabidopsis thaliana*, research on seed size and number is limited by the absence of suitable high throughput phenotyping methods.

**Results:**

We report on the development of a high throughput method for counting seeds and measuring individual seed sizes. The method uses a large-particle flow cytometer to count individual seeds and sort them according to size, allowing an average of 12,000 seeds/hour to be processed. To achieve this high throughput, post harvested seeds are first separated from remaining plant material (dust and chaff) using a rapid sedimentation-based method. Then, classification algorithms are used to refine the separation process in silico. Accurate identification of all seeds in the samples was achieved, with relative errors below 2%.

**Conclusion:**

The tests performed reveal that there is no single classification algorithm that performs best for all samples, so the recommended strategy is to train and use multiple algorithms and use the median predictions of seed size and number across all algorithms. To facilitate the use of this method, an R package (*SeedSorter*) that implements the methodology has been developed and made freely available. The method was validated with seed samples from several natural accessions of *Arabidopsis thaliana,* but our analysis pipeline is applicable to any species with seed sizes smaller than 1.5 mm.

## Background

Seed size is an important plant trait. Seed size affects dispersal and tolerance to abiotic stresses such as deep shade or drought [1]. In addition, seed size and number distributions (i.e. not just the mean values but also the variance around this mean) can be treated as proxies of plant fitness [2, 3] and hence are of interest in evolutionary and ecological studies. Seed size is also an important trait in the quality and market value of cereal grains [4, 5] and in the starting materials industry.

*Arabidopsis thaliana* is a commonly used model plant species due to its ease of cultivation, proliferate propagation, extensive natural variation and availability of genetic tools [6]. Therefore, *Arabidopsis* has often been used in seed biology, to study, for instance, the genetic regulation of seed quality aspects such as dormancy and longevity [7, 8], seed development and how seed size is determined [4, 9]. However, such research is often hampered by the lack of high throughput phenotyping and sorting techniques to quantify seed size distributions [10]. Current techniques often require laborious separation of seeds from other plant materials with the aid of (dissecting) microscopes, followed by the use of pictures or flatbed scanners to digitize the seeds and measure their size by digital image analysis [10–12]. A recent alternative is the *phenoSeeder* platform that allows measurement of the 3D volume and mass of individual seeds, but still requires manual separation of seeds from other plant materials [13].

Seeds of *Arabidopsis* are relatively small, with diameters ranging from 250 μm to 600 μm [10–12], yet they are too large for conventional flow cytometry methods that are typically restricted to particles smaller than 100 μm [14]. Conventional flow cytometry is therefore limited to small biological entities, from the amount of nuclear DNA [15] to pollen grain morphometry [16]. However, with the recent development of large particle flow cytometry (LPFC), the sortable range has increased to 1500 μm [14]. LPFC methodology thus has great potential for quantifying seed traits such as seed numbers and size with higher throughput and improved efficiency compared to current techniques.

Despite the potential of high throughput sorting that can be achieved with LPFC on small seeds such as those of *Arabidopsis,* a naive use of LPFC will overestimate seed count, due to contamination from fragments of broken siliques/seed pods (chaff), dust particles, etc. This can be (partially) overcome by pre-processing samples to remove non-seed material. However, this can be quite laborious and time consuming, and small dust particles will usually remain. These contaminations will consequently result in incorrect statistics (e.g. number of seeds or average seed size). Therefore, a post-sorting method is required to separate non-seed material detected by the flow cytometer from seeds.

If tissue-specific fluorescence labelling can be used, then an accurate distinction between seed and non-seed is feasible with fluorescence detectors built in the LPFC [14]. However, fluorescence labelling is not always possible or desirable as they rely on *e.g.* a fluorescent DNA intercalating dye, or a transgenic modification to express fluorescence-tagged (GFP or RFP) proteins such as in the seeds. This limits the use to either transgenic lines and/or may pose problems if the sorted seeds need to stay viable and undisturbed for follow-up procedures. Alternatively, one may identify the different types of particles in a sample by making use of the optical and geometrical features measured by LPFC, including time-of-flight (TOF, proportional to seed size), optical density, natural auto-fluorescence and high resolution axial profiles of particle optical density [14]. Thus, even if the sample contains dust particles and non-seed plant material, it would still be possible to exclude the non-seed particles in the analysis phase.

We describe here the development of an LPFC-based method to determine seed sizes in an accurate, efficient and high throughput manner. The method combines a coarse and quick sample cleaning procedure based on sedimentation to remove most of the dust and chaff, with LPFC sorting and machine learning classification algorithms. Several machine learning algorithms are compared in their predictive ability to classify seed particles correctly, including both supervised and unsupervised algorithms. To train and evaluate the performance of different algorithms, seeds from five natural *Arabidopsis* accessions (Col-0, Bay-0, Bur-0, An-1 and Lp2-6) as well as dust and chaff particles were manually separated and sorted. To facilitate the use of this method, all necessary computations have been implemented into an R package (*SeedSorter*) that is freely available online at https://github.com/aleMorales/SeedSorter.

## Methods

### Plant growth conditions

Seeds of natural *Arabidopsis thaliana* accessions Col-0 (N1092), Bay-0 (N954), Bur-0 (N1028), An-1 (N944) and Lp2-6 (N22595) were used (Arabidopsis stock accession numbers between brackets, from www.arabidopsis.info). Plants were sown on moist Primasta soil (mixture of potting soil and perlite) and stratified in darkness at 4 °C for four days. After stratification, pots were placed in a climate-controlled room at 21 °C, 70% relative humidity, 120 μmol m^−2^ s^−1^ light intensity (PAR) (measured at rosette height) and a photoperiod of 8 h. When seedlings produced two true leaves (ca. 16 days after sowing), they were transplanted to Jiffy 7c coco pellets (Jiffy Group) that had been previously soaked in lukewarm water and 50 mL of Hoagland’s solution. Additional Hoagland’s solution was applied two, six and eight days after transplanting (10 mL, 20 mL and 10 mL, respectively). The pellets with plants were kept in trays that were watered every other day until the plants set seed and eventually senesced. From each accession, seeds of three individual plants were harvested and processed as described below.

### Protocol overview

**Fig. 1:**
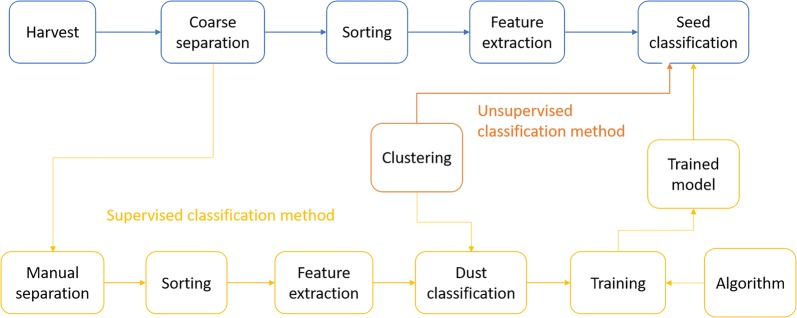
Scheme of the procedure for seed sorting and classification using supervised and unsupervised classification methods. The supervised method requires training of a classification algorithm, for which manual separation of seed samples under a (dissecting) microscope and dust classification via clustering is required. In the unsupervised method, the clustering algorithm is applied directly without prior training (and thus no manually-separated samples are required). Coarse separation involves sieving, sedimentation and drying prior to subsequent sorting performed by the large-particle flow cytometer. Feature extraction calculates indices summarizing the shape of each particle from the high-resolution axial profiles. All the steps after sorting are implemented in the *SeedSorter* R package

In order to avoid blocking the LPFC tubing system and to remove a significant portion of the non-seed particles, the harvested material is first subjected to a coarse separation based on sieving and sedimentation (Fig. 1). This material is then loaded into the LPFC, which sorts all the particles according to their size and records additional optical and geometrical properties of the particles. The output from the LPFC is then processed to extract the main features of each particle that will be used to classify it as either seed or non-seed particles (Fig. 1). Finally, a clustering technique (unsupervised classification method) or a supervised classification algorithm trained on samples containing manually separated seeds is used to classify each particle sorted by the LPFC as seed or non-seed.

Whereas the unsupervised methods can be applied directly, supervised methods require prior training on data where the user assigns which particles need to be considered as seeds and which not. In order to obtain adequate data for training the algorithms, some samples have to be further processed by manually separating the seeds, and the data from sorting these particles through LPFC is then further processed by a clustering method to separate the small dust particles (smaller than 200 μm), that e.g. remain on the surface of the seeds, from the seeds themselves (larger than 200 μm). These two steps result in datasets that per definition should only contain seeds. The classification algorithms can then be trained on the seed-only datasets combined with data from samples with only non-seed plant elements (Fig. 1).

### Seed harvest from the mother plant

When plants started bolting, an Aracon system (Betatech BVBA) was fitted around the plant and kept until the seeds were harvested. The base of the Aracon system collected the seeds that detached after siliques opened while the Aracon tube simultaneously prevented contamination from neighbouring plants, as well as unwanted spreading of the seeds. When the plants were completely senesced, the inflorescence was cut at the base, and all seeds attached to the plant were removed and separated from mother plant tissues, by gently squeezing the dried inflorescence on a sieve (Retsch Gmbh, mesh size: 425 µM), together with the material that was collected in the Aracon base. The collected material was sieved twice thereafter to further clean the sample and then stored in a paper bag. Together, this procedure ensured that virtually all seeds produced by a single mother plant were collected, yet a sizeable fraction of dust and chaff remained.

### Coarse seed separation

A coarse separation protocol was designed to remove most dust and chaff particles that were larger and/or less dense than seeds. The largest elements were first removed with a fine sieve and the remaining material was then transferred to a 50 ml tube with ca. 30 ml of ethanol (96% v/v). Fast sinking seeds were directly collected with a 5 ml Gilson pipette, while less dense particles (dust and chaff) remained floating in the supernatant. Collected seeds were then air-dried on filter paper, and the supernatant ethanol was decanted over a filter paper, folded in a funnel on top of a 500 ml flask. The latter served two purposes. The dust/plant particles in the supernatant became trapped on the filter paper and were collected, dried and used for training and testing the algorithms, while the costly flow-through ethanol could be reused again for subsequent separations. Dried coarse-cleaned seed samples were finally weighed using an ultra-balance.

### Manual seed separation

Seeds were manually separated from chaff on ethanol-drenched filter paper, using a dissecting microscope and preparation needles. The separated seeds were thereafter collected in an Eppendorf tube and allowed to air dry, and finally, when all samples were collected, were completely dried with a SpeedVac concentrator for 30 min. These samples are referred to as ‘manually-cleaned’ in the text and were used for training and performance evaluation of the different classification algorithms.

As the manual separation of seeds could not guarantee the total absence of the smallest dust particles on for instance the surface of the seeds, the particles were further separated after LPFC sorting into two groups by using a K-means clustering technique [17]. Very similar results were obtained by simply defining dust particles as all those particles smaller than 200 μm, as the clusters were clearly separated on either side of this threshold (Additional file 1: Fig. S1).

A limitation of using manually-cleaned seeds is that dust particles may not have the same characteristics as the non-seed particles that result from the coarse cleaning procedure. Therefore, in order to adequately train the supervised classification algorithms, the data from the manually-cleaned samples processed with clustering, was mixed in silico with output from the LPFC for a sample composed of exclusively non-seed particles after the same coarse-cleaning method as for normal samples (see Fig. 1). Thus, the algorithms were trained on samples that resembled in characteristics the coarse-cleaning samples but where all particles were assigned as either seed or non-seed.

### Sorting with large-particle flow cytometer

The BioSorter Large Particle Flow Cytometer (Union Biometrica, Holliston, MA) of the Utrecht University Biology dept. Sorter Facility (https://www.uu.nl/en/research/developmental-biology/sorter-facility) was used for the sorting step. The BioSorter uses easily exchangeable fluidics modules (FOCA) optimized for a particular size range. We used the FOCA 1000 that is optimal for the range of 200− 700 μm. The BioSorter ran on the FlowPilot software (Union Biometrica, Holliston, MA) with the following settings: sample cup pressure: 0.3, diverter pressure: 2.3 and extinction gain: 1. The particles (seeds and non-seeds) were diluted in water and introduced into the system via the sample cup. Between consecutive samples the system was cleaned with water, to avoid cross-sample contamination.

The BioSorter is based on the same working principle as a traditional flow cytometer but with lower pressure and slower flow rates to avoid large shear forces. An air diverter is utilized to dispense samples in a fluid drop into multi-well plates, tubes or stationary receptacles. The heart of the BioSorter, the Fluidics and Optics Core Assemblies (FOCA), is exchangeable in order to analyze, sort and dispense a large size range of samples (from 10 to 1500 µm). The sample travels into a flow cell, where it is surrounded by sheath fluid to focus the sample into the center of the stream. Here, an axial light-loss detector measures the relative axial size of the particle. The larger a particle is, the longer it requires to pass the detector, resulting in the so-called time of flight. With the use of beads of known size, it is possible to calibrate the Biosorter to measure particle sizes. From the total integrated signal of light that is blocked by a particle, the optical density can be determined. A setup of up to three different lasers allow a simultaneous measurement of fluorescence emitted by the particles at three different wavelengths.

### Feature extraction

**Fig. 2:**
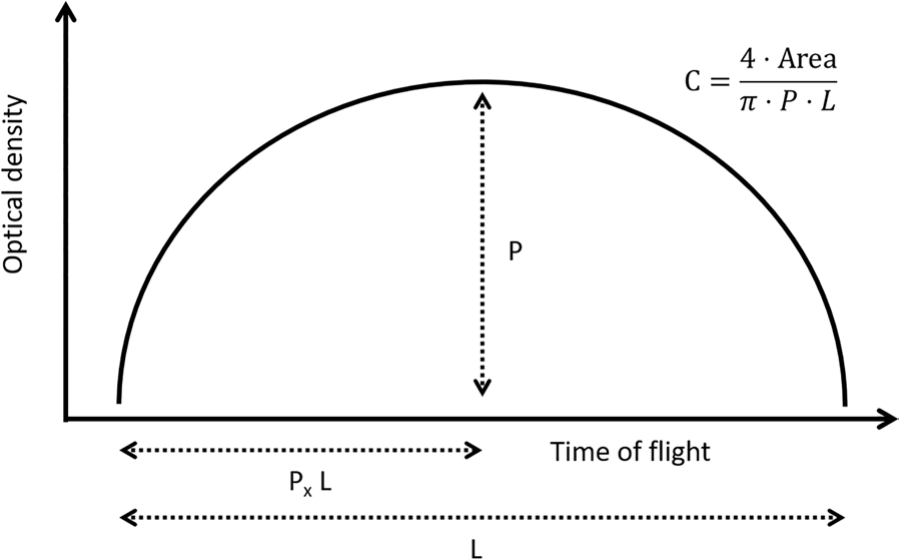
Schematic representation of the profile of a sorted particle. P is the maximum optical density registered by the sensor, P_x_ is the time at which P occurs, relative to the total time-of-flight of the particle (L) and C is the circularity index calculated by comparing the area under the actual profile and the area of the closest elliptical profile

For each sorted particle, the LPFC measured optical density along the main axis of the particle (Fig. 2), fluorescence emission in three different wavebands and the relative axial length of the particle measured as TOF. From these data, a series of features were derived and used by the classification algorithms (Table 1).

**Table 1:** Features calculated for each sorted particle using measurements by the large particle flow cytometer

Feature	Description
Extinction	Integral optical density of the particle
Size	Calculated from Time-of-flight (TOF) using a calibration curve constructed with standard beads (size = 0.18 TOF + 38.32)
rF510	Fluorescence emission in the waveband 497–523 nm relative to average fluorescence emission across all wavebands
rF_543_	Fluorescence emission in the waveband 531–555 nm relative to average fluorescence emission across all wavebands
rF_615_	Fluorescence emission in the waveband 602–628 nm relative to average fluorescence emission across all wavebands
P	Maximum optical density in the optical density profile (Fig. 2)
P_x_	Location within the optical density profile where the maximum occurs (Fig. 2)
C	Circularity index calculated from the optical density profile (Fig. 2)

### Performance evaluation of different algorithms

The following supervised classification algorithms were compared in the analysis: linear discriminant analysis [18], quadratic discriminant analysis [18], logistic regression with regularization via elastic net [19], naive Bayes classifier [20], weighted K-nearest neighbours [21], support vector machine [22], extreme gradient boosting [23], and random forest [24]. In addition, an *ad-hoc* algorithm was created that classified seeds according to a single feature (Extinction, see above) by calculating an optimal threshold that minimized balanced error (see below) using Brent’s method [25]. Furthermore, the K-means clustering algorithm [17] was used as unsupervised classification method to divide the seed sample into two clusters (and the average particle size and number of particles was used to determine which cluster corresponded to seeds).

Three types of performance evaluations were performed on each algorithm:

1. Predictive performance within the seed sample (IntraPlant): The algorithms were trained on a subset of each seed sample and their performance tested on the remaining data from the same seed sample (a sample corresponds to one harvested plant). The subsets were selected randomly using a stratified five-fold cross-validation scheme [26]. This evaluation measures the best performance an algorithm can reach in this methodology as training and predictions are performed on seeds from the same plant.
2. Predictive performance within genotypes (IntraGenotype): The algorithms were trained on each seed sample and predictions were performed on seed from different plants of the same accession (genotype) as the one used for training. This evaluation measures the performance of an algorithm when extrapolating across plants and therefore represents the most common scenario in application of the methodology (i.e. predictions are made for plants of the same genotypes and grown under the same conditions).
3. Predictive performance across genotypes (InterGenotype): The algorithms were trained on each seed sample and predictions were performed on seeds from different plants and different accessions from the one used for training. This evaluation measures the performance of the algorithms in situations where predictions are made on plants that may have different seed traits (e.g. different size and optical density), compared to the ones used for training the algorithms.

The datasets used for training and evaluation of the performance of the algorithms did not have the same amount of seeds and non-seed particles. This imbalance between the two classes of particles can lead to suboptimal predictive performance, as the algorithms will emphasize correct classification of the majority class, at the expense of the minority class (which was always the class containing seeds in the training datasets). To avoid this problem, random oversampling (i.e. random sampling without replacement) of the minority class by a factor of ten (which was the average ratio of class sizes) was applied to each sample used for training. In addition, specific algorithms required further processing of the raw data as well as tuning of hyperparameters (see Additional file 1).

As performance criteria to rank the algorithms, the ‘balanced error rate’ (BER, average of the mis-classification error for seed and non-seed particles, where the misclassification error is calculated as the fraction of particles that are not classified correctly in each class), was used. The use of BER allows avoidance of over-optimistic estimates as false positives and false negatives contribute equally to the error, even when the two classes are highly imbalanced, whereas a single mis-classification error will be biased towards the class that is more abundant in the sample.

### Classification of coarse-cleaned samples

To illustrate the use of the method on coarse-cleaned samples, additional plants for each accession (grown under the same conditions as the plants use for training and performance evaluation) were harvested and subjected only to the coarse-cleaning procedure. Then, the supervised algorithms trained on each accession using all the manually-cleaned data available were used to classify the sorted particles in the coarse-cleaned samples. For each sample, the number of seeds and median seed size was calculated. The variation in predictions of these quantities across all algorithms was quantified as relative changes with respect to the median values across algorithms. Also, the variation across accessions in median seed size and the relationship between sample weight and number of seeds was analysed using median predictions across all algorithms.

### SeedSorter package

All the analysis was performed in the R programming language [27] and the machine learning pipelines were constructed with the *mlr* package [28]. To facilitate the application of this approach to new datasets, an R package (*SeedSorter*) was developed that implements the machine learning pipelines and all the data pre-processing tasks. All the supervised and unsupervised classification algorithms were included in the package as well as functions to evaluate the performance of the algorithms on different seed samples.

The R package *SeedSorter* is available online at https://github.com/aleMorales/SeedSorter. The results presented in this paper were all obtained using this R package. The R scripts and data required to reproduce these results can be obtained at https://github.com/aleMorales/SeedSorterPaper. An introductory tutorial to the use of the SeedSorter package is provided as Additional file 1.

## Results

### Manually-cleaned samples

Despite the manual separation, the clustering on manually-cleaned samples identified between 16.6% (accession Lp2-6) and 39.1% (accession An-1) of the detected particles as non-seed (dust). The samples were also processed by assuming that all particles larger than 200 μm were seeds and the smaller particles were dust. Both approaches (clustering and separation by a fixed threshold) agreed fairly well and the fraction of particles where the two approaches did not agree ranged from 0.7% (Lp2-6) to 4.1% (An-1). The larger discrepancy for An-1 can be explained by the smaller seed size as compared to Lp2-6, which resulted in a relatively larger overlap between the seed and non-seed classes (Additional file 1: Fig. S2). The training of the classification algorithms was performed with the datasets processed by the clustering technique.

**Fig. 3:**
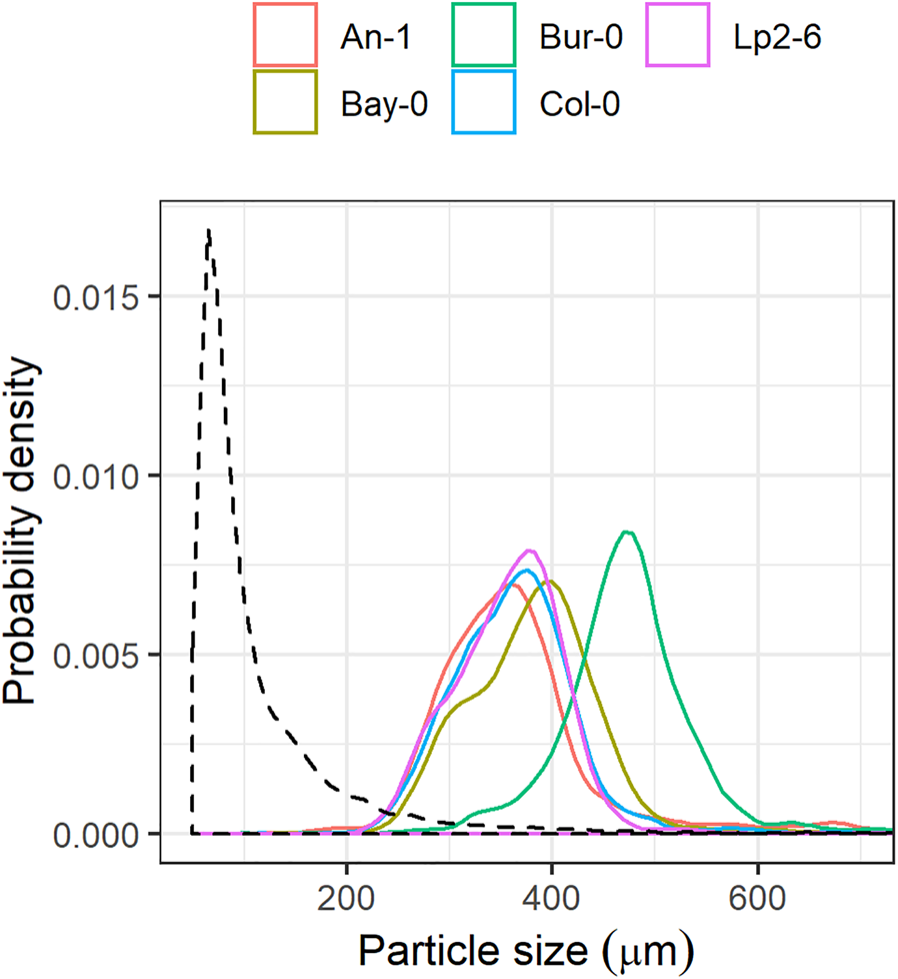
Distribution of particle sizes of the manually-cleaned seed samples after clustering was applied to remove the dust particles. Coloured solid lines indicate seeds of different accessions; black dashed lines indicate added non-seed particles (i.e. dust)

After clustering the manually-cleaned samples, an average of 1420 seeds per sample were identified, with a median seed size of 363 μm and 95% of the seeds having a size between 255 μm and 665 μm (Fig. 3). Most accessions had similar seed size distributions (medians between 306 μm for An-1 and 359 μm for Bay-0), except for Bur-0 that a had a median seed size of 451 μm.

### Performance evaluation of algorithms

The samples used for training the algorithm had, on average, 9800 non-seed particles per sample, with a median size of 84.2 μm, but about 10% of these particles were larger than 200 μm (Fig. 3). This implies that, unlike the manually-cleaned samples that were only contaminated with small-particle dust, a significant portion of the non-seed particles that were not removed by the coarse cleaning were of similar size to the seeds, justifying the inclusion of additional features to distinguish between seeds and non-seeds.

**Fig. 4:**
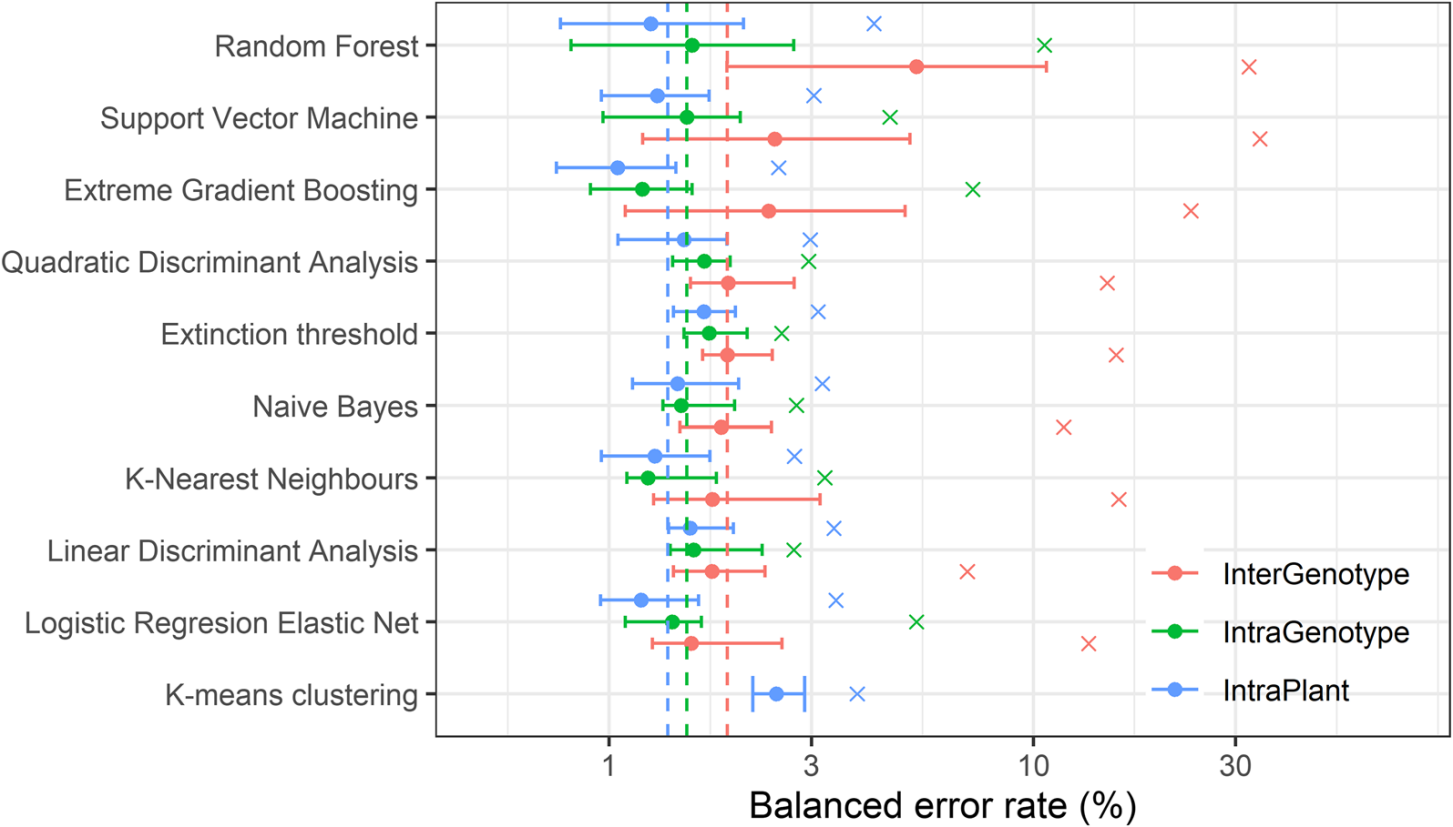
Balanced error rate (BER) of predictions within plant (IntraPlant), across plants from the same accession (IntraGenotype) and across plants from different accessions (InterGenotype) for the different algorithms compared in this study. The solid circles indicate the median of all predictions for a given algorithm and type of comparison. The error bars indicate the interquartile range (i.e. between percentiles 25% and 75%) of these predictions. The crosses beyond the error bars indicate the BER achieved for each algorithm and type of comparison. As K-means clustering is an unsupervised classification method, only IntraPlant predictions are shown for this method. Supervised classification algorithms are sorted according to their median performance across accessions (InterGenotype), from worst to best. Dashed vertical lines represent the median BER across all predictions by all supervised classification algorithms for each type of comparison. Three plants were used for each accession for a total of 15 plants

When the predictive performance of algorithms was tested with data obtained from the same plant as used for training (IntraPlant), the best performing algorithm was Extreme Gradient Boosting (Fig. 4), with a median BER of 1%, while the worst performance was by the Extinction threshold algorithm with a median BER of 1.7%.

When algorithms were evaluated with data from other plants, from the same (Fig. 4; IntraGenotype) or different accessions (Fig. 4; InterGenotype), the BER increased for all the algorithms, being worst when classifying seeds from a different accession to the one used for training. Thus, the median BER for predictions within and across accessions increased to 1.5% and 1.9%, respectively. Some algorithms (e.g., random forest, support vector machine and extreme gradient boosting) were particularly affected by the use of data from different accessions for training and performance evaluation (InterGenotype), whereas others (e.g. logistic regression, linear discriminant analysis) were more robust. Performance was relatively low when making predictions for Bur-0, when the algorithm had been trained on a different accession (Fig. 1; Additional file 1: Fig. S3), which is likely due to the different seed characteristics of this accession as compared to the other four accessions (Fig. 3). In most cases, the highest BER for predictions across accessions were above 10% and reached as high as 32% (Fig. 4), whereas predictions within accessions always had a BER below 12%.

The variation across datasets in predictive performance was particularly large for random forest, support vector machine and extreme gradient boosting, when making predictions across accessions (InterGenotype; 7.7%, 1.8 and 4.0%, respectively). This led to large errors in some cases (e.g. the largest BER was 34% with a support vector machine trained on data from Lp2-6 and making predictions for Bur-0).

The unsupervised classification algorithm (K-means clustering, Fig. 4) performed worse than all supervised classification algorithms, when these were evaluated with data from the same plant or accession, with a median BER of 2.5%. It only performed better than some algorithms when predictions were made for plants from other accessions than the one used for training. On the other hand, the largest BER for the K-means clustering algorithm was only 3.8%, smaller than the maximum error rates for any other algorithm in the comparison.

### Seed classification for coarse-cleaned samples

The different algorithms made, on average, similar predictions of number of seeds and median seed size for the coarse-cleaned samples (Additional file 1: Fig. S4). For most algorithms and samples, the relative difference in number of seeds and median seed size with respect to the overall median across algorithms was smaller than 10% and 2% respectively (Additional file 1: Fig. S4). The algorithms that deviated the most from the overall trend were the support vector machine, random forest, extreme gradient boosting and the Extinction threshold algorithm (Additional file 1: Fig. S4). However, in all cases there were worst-case scenarios where relative differences with respect to the median across all algorithms exceeded 10% (for the median seed size) or 50% (for the number of seeds).

**Fig. 5:**
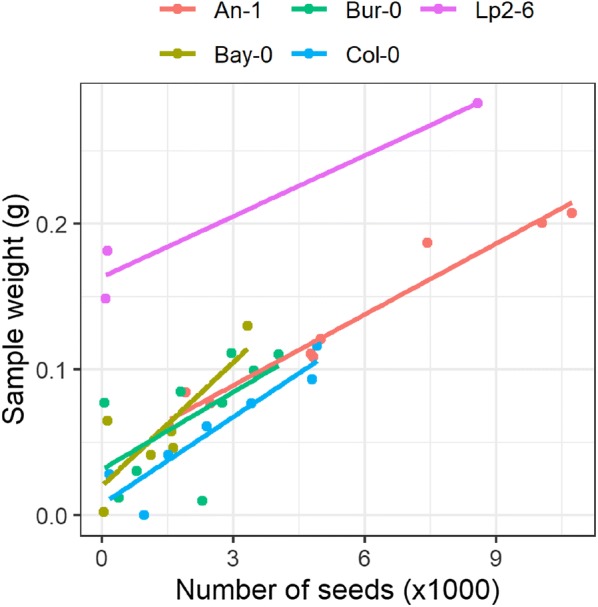
Weight of each sample (weight of all seeds from a single plant including non-seed particles that remain after coarse cleaning) versus the number of particles classified as seeds in the sample, based on the median prediction across all algorithms for different Arabidopsis accessions

**Fig. 6:**
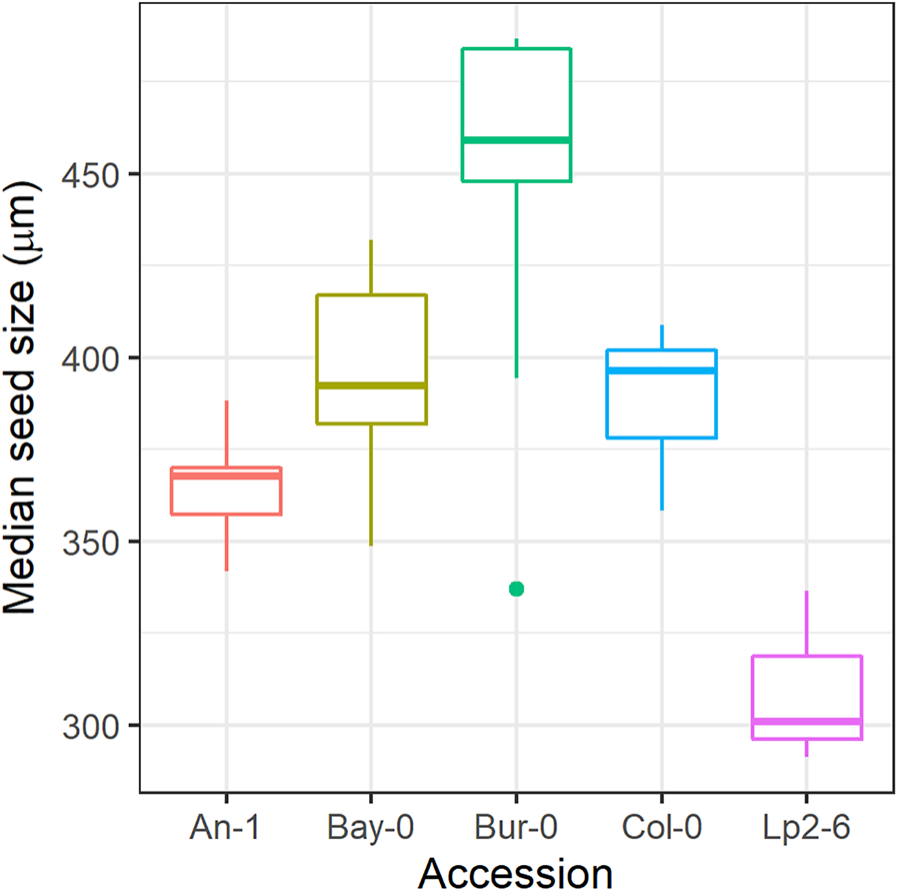
Boxplots of median seed size (calculated from time-of-flight) in the coarse-cleaned samples for each of the five accessions (same data as for Fig. 5). For each sample and supervised classification algorithm, the median size of the particles classified as seeds was calculated, and then the median of these medians across all algorithms for each plant was then computed and used to construct the boxplots

As expected, the overall weight of the samples (i.e. all seeds derived from one plant, after coarse-cleaning) was highly correlated with the predicted number of seeds for the same plant (Fig. 5). The predicted average particle weights (calculated as the slope of the linear regression between sample weight and number of seeds, Fig. 5) varied from 13.9 ± 3.3 μg (Lp2-6) to 28.3 ± 9.8 μg (Bay-0), with an average of 19.2 ± 3.3 μg. On average, each plant had about 3000 seeds, although there was a wide range of values within and across accessions (Fig. 5). The median seed size was predicted to be fairly conserved with the largest value for Bur-0 and the lowest for Lp2-6 (Fig. 6), in agreement with results from the manually cleaned samples (Fig. 3).

The discrepancy between median size and average particle weight (especially in the case of Bur-0 and Lp2-6) may be the result of different levels of contamination by non-seed particles or by different seed densities. However, the strong linear relationship between sample weights and seed number (Fig. 5) suggests that the contribution of non-seed particles to the overall sample weight is likely small.

## Discussion

### The applicability of the method

In this study, we present a high throughput LPFC-based method to count seeds and quantify morphological features such as individual seed sizes. The method relies on classification algorithms to distinguish between seeds and non-seed particles sorted by large particle flow cytometry (LPFC). The performance of different algorithms was compared for different scenarios that emulate the different contexts in which the method is likely to be used in practice.

In our laboratory, the average time required for coarse-cleaning of the seeds harvested per plant was approximately 5 min, whereas the time required to sort the sample through the LPFC was 10 min. This means an average throughput of 4 plants/hour. Assuming an average of 3000 seeds per plant (Fig. 5), this results in a throughput of 12,000 seeds/hour. Using the *SeedSorter* R package, the training of an algorithm can take from seconds to minutes (depending on the algorithm used and computational power available), but the time needed to make a prediction in our laboratory was in the scale of seconds or less. Therefore, the computational component does not add much time to the overall procedure.

We want to emphasize that the procedure described in this study does not only apply to *Arabidopsis* but can be of interest for the study of many plant species with seeds in the range of 100 μm to 1500 μm (e.g. tobacco, onion, carrot, rapeseed, orchids, *Rumex spp* etc.) as well as megaspores from lycopods and ferns [29, 30]. Westoby et al. [31] reported that 10–20% of the plant species in five different ecosystems had seed weights lower than 100 μg/seed (which are likely to be smaller than 1500 μm), so our methodology may also be useful for evolutionary/ecological studies. The lowest size for which our methodology is useful is determined by the ability to reliably distinguish seeds from dust particles (though some distinction may be possible with optical density), whereas the largest size is limited by the LPFC technology used.

### Performance evaluation of algorithms

Despite that manual separation of seeds could not exclude all non-seed particles, because of the presence of dust in the samples, the dust particles were easily identified either by clustering or by applying a threshold based on particle size. We are confident in this separation, as non-seed particles that are larger than 200 μm would have been undoubtedly recognized during the manual cleaning process otherwise. Moreover, seeds of *Arabidopsis* are unlikely to be smaller than 200 μm [10–12]. Also, the range of seed sizes predicted agrees with previous reports for Arabidopsis [10–12].

The manual separation led to similar seed size distributions for all accessions except for Bur-0, that had larger seeds. This is in agreement with previous research on natural variation in seed size in *Arabidopsis* [32]. These results were confirmed with the coarse-cleaned samples, with Bur-0 also having larger seed sizes, although the variation in the other accessions was larger than in the manually-cleaned samples (Fig. 6).

Interestingly, the differences in median seed size across accessions (Fig. 1) were not proportional to differences in average particle weight (Fig. 1), although our calculated average particle weights and the number of seeds per plant were quite similar to previously reported seed weights and number in *Arabidopsis* [33, 34]. The strong correlation between sample weights and seed number (Fig. 5) indicated that this discrepancy was not caused by the contribution of non-seed particles. Potential explanations would include differences in seed shape resulting in different seed volumes for the same seed axial length or a different seed density (i.e. mass/volume) due to changes in seed composition. Seed shape in *Arabidopsis* is known to be regulated by the phytohormones brassinosteroid [11] and ethylene [35] and different mutations can break the relationship between seed weight and length by varying the length/width ratio [11], so there likely is natural variation in seed shape in *Arabidopsis*. Regarding possible changes in seed density, Janhke et al. [13] measured the mass and volumes of individual seeds of *Arabidopsis* and reported a variation of 10% in seed density across three natural accessions*.*

Our results indicate that the optimal algorithm depends on whether or not one has access to manually-cleaned samples for the same accession and growth conditions as the samples of interest. For this decision, the performance in the IntraPlant scenario is not relevant, as one does not need to make predictions for a plant for which manual separation has already been performed on the whole harvest. Rather it represents the maximum performance that can be achieved given the features available per particle and the uncertainty associated with the identification of dust particles in the manually-cleaned samples.

All algorithms performed worse when making predictions across accessions compared to within accessions (Fig. 3, Additional file 1: Fig. S2), but some algorithms were particularly sensitive (especially random forest, support vector machine, and the extreme gradient boosting). Interestingly, the extreme gradient boosting had the best performance when making predictions for data coming from the same plant or same accession but was the third worst performing algorithm in making predictions across accessions. This pattern reveals that these algorithms were capable of capturing subtle relationships among features that are very specific to a particular plant or accession but are not necessarily conserved across accessions. On the other hand, other algorithms that may be less efficient at capturing these subtle relationships (e.g. regularized logistic regression, linear discriminant analysis, naive Bayes or the Extinction threshold algorithm) are more robust against changes in the underlying patterns and are therefore more accurate when extrapolating to other accessions. The ability to capture subtle differences between accessions could potentially open ways to develop protocols for efficient screening of (natural) variation in seed traits e.g. seed quality or uniformness traits.

The performance of the different algorithms was also reflected in the predictions for coarse-cleaned samples (Additional file 1: Fig S4). Algorithms that had high predictive power (within accessions) made similar predictions, whereas random forests and support vector machines deviated more strongly from the average behaviour. In general, there was better agreement among algorithms in predicting the median seed size than in the number of seeds. However, in both cases, there were significant outliers where an algorithm will deviate strongly from the predictions made by most other algorithms (Additional file 1: Fig. S4), in a similar fashion to the outliers detected in the evaluation of performance (Fig. 4). Therefore, to avoid introducing significant biases in the predictions for particular samples, we recommend using multiple algorithms and calculate the median prediction for each quantity across all algorithms as we did in this study (Figs. 5, 6).

### Recommendations based on performance evaluation

We recommend that when manually-cleaned samples are not available for the same accession and treatment as the samples of interest, and when the median performance is used as criterion for selection, the relatively simpler algorithms such as regularized logistic regression, linear discriminant analysis, K-nearest neighbours, naive Bayes or the Extinction threshold algorithm should be used. The performance of these algorithms is similar, and they are computationally efficient. When manually-cleaned samples of the same accession and treatment are available for training, then extreme gradient boosting is also a good choice (in addition to the algorithms above). Finally, our analyses suggest not to use quadratic discriminant analysis (as the linear version always performed better) or random forest or support vector machine, as these algorithms are both computationally intensive and do not perform as well as the other algorithms.

Although the recommendations above are based on the median predictive performance, the distribution of error rates was highly skewed and, especially when making predictions across accessions, maximum error rates were much higher than the median (Fig. 4). These worst-case scenarios represent situations where the patterns in the data differ substantially between datasets for training and performance evaluation, leading to biased prediction by specific combinations of algorithms and samples.

In the absence of manually-cleaned samples, predictions can be obtained by using the unsupervised classification algorithm (i.e. K-means clustering). Another advantage of this method is that its error rates are constrained within a relatively narrow range (Fig. 4). The reason why clustering will perform relatively well in any case is that the prediction is always based on the data being classified, meaning the algorithm did not learn any particular pattern from other data. Of course, the trade-off is that the BER will be, on average, higher than for supervised methods. Whether a 2.5% error (Fig. 4) is acceptable or not depends on the specific context of the analysis (i.e. other sources of experimental error, number of replicates, size of effect being quantified, etc.), so a general recommendation cannot be given in this context. Another limitation to the direct application of clustering is that one must make assumptions regarding which of the two resulting clusters corresponds to seeds.

## Conclusions

We conclude that it is not possible to define a single optimal algorithm for all possible scenarios. Furthermore, it is probable that the algorithms will perform differently with different species or growth conditions and that different choices should be made. For that reason, the *SeedSorter* R package includes interfaces that aid future users in comparing the different algorithms, calculating the error rates and visualizing the results (i.e. producing figures like Fig. 3 or Additional file 1: Fig. S2) on new data, without requiring any expertise on machine learning. Furthermore, the source code supporting this study (see section “Methods”) can be used as template for future analysis of new experimental data, and the raw data is also provided to facilitate reproduction and comparison.

The system could be adapted to classify other types of particles depending on whether the features captured by the flow cytometer are sufficient to distinguish among the different classes of particles and, while currently the *SeedSorter* package only performs binary classification, most of the algorithms it relies on can be extended to multiclass classification.

Although the BioSorter is capable of dispensing samples into multi-well plates, tubes or stationary receptacles by an air diverter, the here-proposed methods are explicitly meant as post-sorting analytical tool to be employed after the physical processing of the seeds by the machine. To our knowledge, implementing the classification algorithms in the decision making algorithms of the Biosorter is currently not possible.

The methodology proposed in this study was able to identify accurately the seeds in the different samples, with relative errors below 2% for most classification algorithms. There was not one algorithm that performed best for all samples, so the recommended strategy is to train and use multiple algorithms and use median predictions of seed size and number. To enable and facilitate the use of this method, an R package (*SeedSorter*) that implements the methodology has been developed and made freely available in an online repository located at https://github.com/aleMorales/SeedSorter. The proposed methodology is useful for quantitative studies on seed size and number in small-seeded species.

## Supplementary information

**Additional file 1.***SeedSorter* user tutorial, additional methods, additional figures.

## Data Availability

The datasets and R script supporting the conclusions of this article are available in the SeedSorterPaper repository, https://github.com/aleMorales/SeedSorterPaper.

## Abbreviations

BER: Balanced error rate
LPFC: Large particle flow cytometry
TOF: Time-of-flight
