# Supplementary material for: A high throughput method for quantifying number and size distribution of Arabidopsis seeds using large particle flow cytometry
Source: Plant Methods. 2020 Mar 2;16:27. doi: 10.1186/s13007-020-00572-x (PMC7053093; doi:10.1186/s13007-020-00572-x)
Supplement: Supplementary file 1 — Additional file 1.SeedSorter user tutorial, additional methods, additional figures. [file 13007_2020_572_MOESM1_ESM.docx]

# Additional file 1

# Hyperparameter tuning and data transformation

The manually-cleaned data was scaled and centred prior to training the following algorithms: support vector machine, regularized logistic regression and K-nearest neighbours. In the case of quadratic discriminant analysis, the algorithm was trained on principal components calculated from the data rather than the original features. The same transformations (either scaling or principal components transformation) with the same parameters were applied to all the data for which predictions were made (both manually-cleaned and coarse-cleaned).

For some of the supervised algorithms, hyperparameters were tuned during the training. The tuning was implemented with the evolutionary optimization algorithm CMA Evolution Strategy^[[1]](#footnote-1)^, in order to find the hyperparameters that minimized the average predictive BER. In order to calculate this error rate, five-fold cross-validation was applied to the data being used to train the algorithms. This means that in the IntraPlant evaluation of performance of those algorithms that required hyperparameter tuning, a 2-level nested stratified resampling scheme was used (the inner re-sampling used for hyperparameter tuning, the outer one for evaluating predictive performance on data not used for training).

The hyperparameters tuned per algorithm were as follows:

- Extreme gradient boosting: The hyperparameters tuned were the number of boosting rounds, the fraction of original data used in each boosting round, the maximum depth of the decision tree constructed in each boosting round and the parameters α, η and λ that control overfitting of the procedure.
- Support vector machine: The hyperparameters tuned were the cost of constraint violations (i.e. the *C* constant) and the inverse kernel width for the radial basis kernel function.
- Logistic regression regularized with elastic net: The hyperparameters tuned were the elastic mixing parameter (i.e. mixing of lasso and ridge penalty terms) and the value of the penalty term for predictions.
- K-Nearest neighbours: The hyperparameters tuned were the number of neighbours to be used by the algorithm.

# Introduction to SeedSorter

## Introduction

SeedSorter is a package that facilitates the use of supervised and unsupervised classification algorithms to discriminate between seeds and non-seed plant particles that have been sorted with an Union Biometrica Biosorter large particle flow cytometer. This tutorial introduces the main functions of SeedSorter to train algorithms, tune hyperparameters, quantify prediction performance with error indices and perform predictions. A real life example of a more complex workflow can be found at <https://github.com/AleMorales/SeedSorterPaper>.

The only dependency for this tutorial is the SeedSorter package, which can be loaded into a new R session as follows:

library(SeedSorter)

## Processing raw data

For each sorted sample, two types of files from the large particle flow cytometer are expected: a file with properties (such as time of flight or integral fluorescence) measured for each particle and a file with the complete profile of optical density of each particle. SeedSorter needs to know the locations of these two files associated with a sample.For the purpose of this tutorial, we will download the files from the Github repository mentioned in the above to a local folder temporary:

dir.create("temporary")
download.file(url = "https://raw.githubusercontent.com/AleMorales/SeedSorterPaper/master/Input/Training/An1_A.txt", destfile = "temporary/An1_A.txt")
download.file(url = "https://raw.githubusercontent.com/AleMorales/SeedSorterPaper/master/Input/Training/An1_A_prf.txt", destfile = "temporary/An1_A_prf.txt")

The first step is to process the files with profile data in order to extract features that are useful for classification (rather than working with the raw profiles). This is achieved with the processFile function. This function will create a new file with the extension *fst* (from the fst package). The processFile function just takes the name of the file with the profile of optical density as input:

processFile(file = "temporary/An1_A_prf.txt")

We are going to need two more samples for this tutorial:

- A second sample of manually-separated seeds from the same genotype.
- A sample with only non-seed plant material that has been exposed to the same coarse-cleaning procedure as other samples.
- A sample with an unknown mix of seeds and non-seed plant material as example of unlabelled sample for which we want to make predictions.

The downloading and processing of these additional files is straightforward:

# Download and process second seed sample
download.file(url = "https://raw.githubusercontent.com/AleMorales/SeedSorterPaper/master/Input/Training/An1_B.txt", destfile = "temporary/An1_B.txt")
download.file(url = "https://raw.githubusercontent.com/AleMorales/SeedSorterPaper/master/Input/Training/An1_B_prf.txt", destfile = "temporary/An1_B_prf.txt")
processFile(file = "temporary/An1_B_prf.txt")

# Download and process non-seed plant material sample
download.file(url = "https://raw.githubusercontent.com/AleMorales/SeedSorterPaper/master/Input/Training/waste.txt", destfile = "temporary/waste.txt")
download.file(url = "https://raw.githubusercontent.com/AleMorales/SeedSorterPaper/master/Input/Training/waste_prf.txt", destfile = "temporary/waste_prf.txt")
processFile(file = "temporary/waste_prf.txt")

# Download and process unlabelled sample (this file is already processed in the online repository)
download.file(url = "https://raw.githubusercontent.com/AleMorales/SeedSorterPaper/master/Input/Application/An-1/156_2_109_01_extra.txt", destfile = "temporary/unlabelled.txt")
download.file(url = "https://raw.githubusercontent.com/AleMorales/SeedSorterPaper/master/Input/Application/An-1/156_2_109_01_extra_ch0_prf.fst", destfile = "temporary/unlabelled_prf.fst", mode = "wb")

## Preparing trainig dataset

In order to train an algorithm we need two types of sorted samples:

- A sample that only contains seeds which has been obtained through manual separation under a dissecting microscope.
- A sample that only contains non-seed plant material that has been subject to the same cleaning procedure as normal samples.

Each sample will have two files associated to it, as explained above. The first step is to load the data and label the different particles as seed or waste (non-seed) particles. This is achieved with the getTrainingData function:

data_train = getTrainingData(main_file = "An1_A.txt",
 profile_file = "An1_A_prf.fst",
 main_waste_file = "waste.txt",
 profile_waste_file = "waste_prf.fst",
 datadir = "temporary", clean = TRUE)

Note that we specify the names of each of the four files as well as the directory where these files are stored (argument datadir). It is important to assign the correct file to each argument to ensure that labelling is done correctly. Also, remember to use the files with fst extension for the profile data. There is an additional argument (calibration) that specifies the calibration coefficients to translate time of flight into particle size, but in this case we are relying on the default from SeedSorter (check help documentation on getTrainingData for details).

The last argument (clean) indicates whether the samples assumed to only have seeds should be cleaned by removing very small dust particles (usually < 100 μm) that are not detectable during manual separation and often stick to the surface of the seeds prior to sorting. This is achieved through the separation of the data into two groups via k-means clustering and retaining the group with higher average particle size. Always use clean = TRUE unless is is certain that there are no small dust particles.

The result of getTrainingData is a table with all the features that can be used to training the classification algorithms, plus the class of particles (S = seed particle, W = waste or non-seed particle):

head(data_train)

## # A tibble: 6 x 8
## Extinction rGreen rYellow P Px C Size Class
## <dbl> <dbl> <dbl> <dbl> <dbl> <dbl> <dbl> <chr>
## 1 18.6 0.730 0.649 3764 0.261 0.697 90.9 W
## 2 20.7 0.818 0.614 3176 0.438 0.943 94.6 W
## 3 34.3 0.75 0.482 5052 0.490 0.867 116. W
## 4 6.74 0.571 0.429 1344 0.362 0.919 62.7 W
## 5 21.6 0.303 0.686 2688 0.387 0.834 103. W
## 6 2.64 1.2 0.6 380 0.136 0.752 49.4 W

In order to train the algorithms on these data, we first need to convert them into a *classification task* as implemented by the mlr package (which is the machine learning backend on which SeedSorter runs). This is achieved with the createTrainingTask function:

task_train = createTrainingTask(data = data_train)

## Training an algorithm

Training an algorithm on a classification task is as simple as calling the trainAlgorithm function, passing the name of the algorithm:

model = trainAlgorithm(algorithm = "xgboost", task = task_train, osw.rate = 3.5)

In this case we have trained the XGBoost algorithm with the data loaded earlier. The list of classification algorithms that can be trained in SeedSorter can be retrieved from the help documentation of the trainAlgorithm function. The argument osw.rate indicates the rate of oversampling to be performed for the smallest class. This is required to avoid biasing the model when trained on an unbalanced dataset. We can estimate the amount of oversampling needed by looking into the description of the classification task:

task_train

## Supervised task: cleanseeds
## Type: classif
## Target: Class
## Observations: 3559
## Features:
## numerics factors ordered functionals
## 7 0 0 0
## Missings: FALSE
## Has weights: FALSE
## Has blocking: FALSE
## Has coordinates: FALSE
## Classes: 2
## S W
## 788 2771
## Positive class: S

The last lines indicate the number of particles in each category and from this we can derive the need to oversample the minority class by 3.5 (the oversampling rate does not have to be very exact; small imbalances will not cause trouble).

## Testing the trained algorithm

After training an algorithm we always want to test how accurate the model is in making predictions on new samples. In order to be able to test the predictive performance of the model, this sample also needs to be labelled (i.e. the dataset for testing a model should have the same features as for training). Like before, the original raw data needs to be combined using the getTrainingData function:

data_test = getTrainingData(main_file = "An1_B.txt",
 profile_file = "An1_B_prf.fst",
 main_waste_file = "waste.txt",
 profile_waste_file = "waste_prf.fst",
 datadir = "temporary", clean = TRUE)
task_test = createTrainingTask(data_test)

And making predictions with the model for this second sample is acomplished with the function testModel:

model_test = testModel(model, task_test)

The predictive performance of the model can be retrieve from the returned object as:

model_test$error

## ber mmce
## 0.0007217611 0.0011239112

Where ber stands for balanced error rate and mmce is the mean misclassification error. Note that both indices report very different values, in this case the balanced error rate is more reliable as the data sample is unbalanced.

## Hyperparameter tuning

The XGBoost algorithm (and others supported by SeedSOrter) can benefit from hyperparameter tuning, that is, from tuning the settings of the algorithm to improve predictive power. This can be achieved by using the function tuneAlgorithm rather than trainAlgorithm:

tuned_model = tuneAlgorithm(algorithm = "xgboost", task = task_train, osw.rate = 3.5, maxiter = 15L, lambda = 15L)

The optimal settings for the algorithm is calculated with an evolutionary optimization algorithm (CMA-ES) which settings are controlled by maxiter (maximum number of iterations) lambda (number of offspring per generation). To avoid overestimation of predictive performance, the data is split into 5 subsets of equal size using a stratified cross-validation technique.

We can now test the model on the second sample and quantify the prediction error and confirm that these are lower that for the original non-tuned model:

tuned_model_test = testModel(tuned_model, task_test)
cbind(`non-tuned` = model_test$error, tuned = tuned_model_test$error)

## non-tuned tuned
## ber 0.0007217611 0.0003608805
## mmce 0.0011239112 0.0005619556

## Predictions on unlabelled samples

Once we have trained and tested an algorithm to quantify its predictive performance, we can use the trained model to make predictions for new samples of unlabelled data (i.e. samples for which manual separation was not applied). The procedure to prepare the data for the model is somewhat different to training and testing. We use the getPredictionData function and pass the two files associated to the unlabelled sample:

unlabelled_data = getPredictionData(main_file = "unlabelled.txt",
 profile_file = "unlabelled_prf.fst",
 datadir = "temporary")

The prediction for unlabelled data is then performed with the classifySeeds function:

prediction = classifySeeds(model = tuned_model, data = unlabelled_data)

Obviously, we cannot calculate the prediction errors as we do not know the true nature of each particle. However, we can extract the class predicted for each particle from the returned object and append it to the unlabelled data:

predicted_class = as.data.frame(prediction)
unlabelled_data = transform(unlabelled_data, prediction = predicted_class$response)

We can now see that the predicted class has been added to each row:

head(unlabelled_data)

## Extinction rGreen rYellow P Px C Size prediction
## 1 6.48 0.46153846 1.6153846 1088 0.4655172 0.8994030 62.745 W
## 2 530.00 0.01124297 0.3185509 41420 0.3087349 0.8909932 286.965 S
## 3 483.00 0.01684239 0.1774613 46936 0.5268199 0.9221998 234.425 S
## 4 6.37 0.17142857 0.4285714 2512 0.3260870 0.8724212 58.305 W
## 5 7.84 0.70588235 0.5294118 4244 0.4130435 0.8834883 58.305 W
## 6 10.20 0.07526882 0.9569892 2520 0.3974359 0.8807999 70.145 W

And we can use this to calculate the number of particles in each category:

table(unlabelled_data$prediction)

##
## S W
## 3268 1662

Retrieve the median size of seeds in the sample:

median(subset(unlabelled_data, prediction == "S")$Size)

## [1] 340.245

Or plot the distribution of seed sizes:

plot(density(subset(unlabelled_data, prediction == "S")$Size), xlab = "Size", main = "")


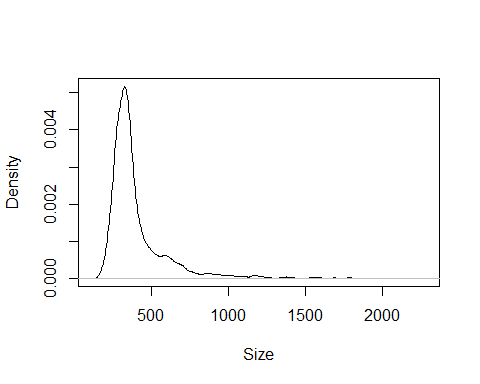


# Supplementary Figures


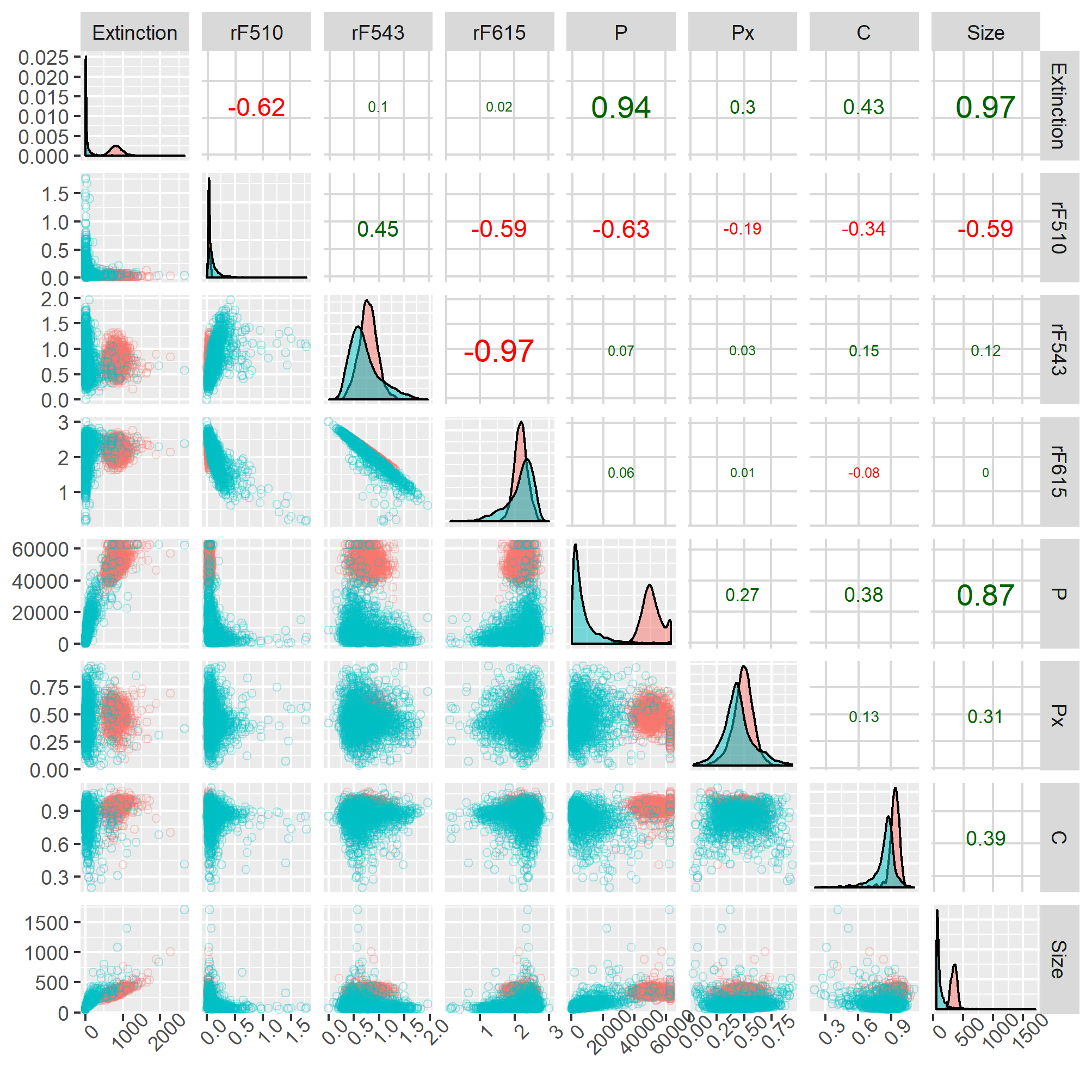


Figure S1: Matrix of scatter plots (lower/left panels) marginal distributions (diagonal panels) and Spearman correlation coefficients (upper/right panels) of all the features considered for the classification, coloured by the type of particle (seeds in red and non-seeds in blue). To facilitate visualization, a randomly generated subset of all the data for accession Col-0 was selected to make this graph (the full data was used in the analysis described in the main text). rF510, rF543 and rF615 correspond to the relative fluorescence emission in the wavebands 497 – 523 nm, 531 – 555 nm and 602 – 628 nm, respectively, P is the maximum signal from the particle profile, Px is the relative location in the particle profile where the maximum signal occurs and C is the circularity index calculated from the particle profile. The size of the correlation coefficient is proportional to its value, whereas the colour indicates positive (green) or negative (red) correlation.


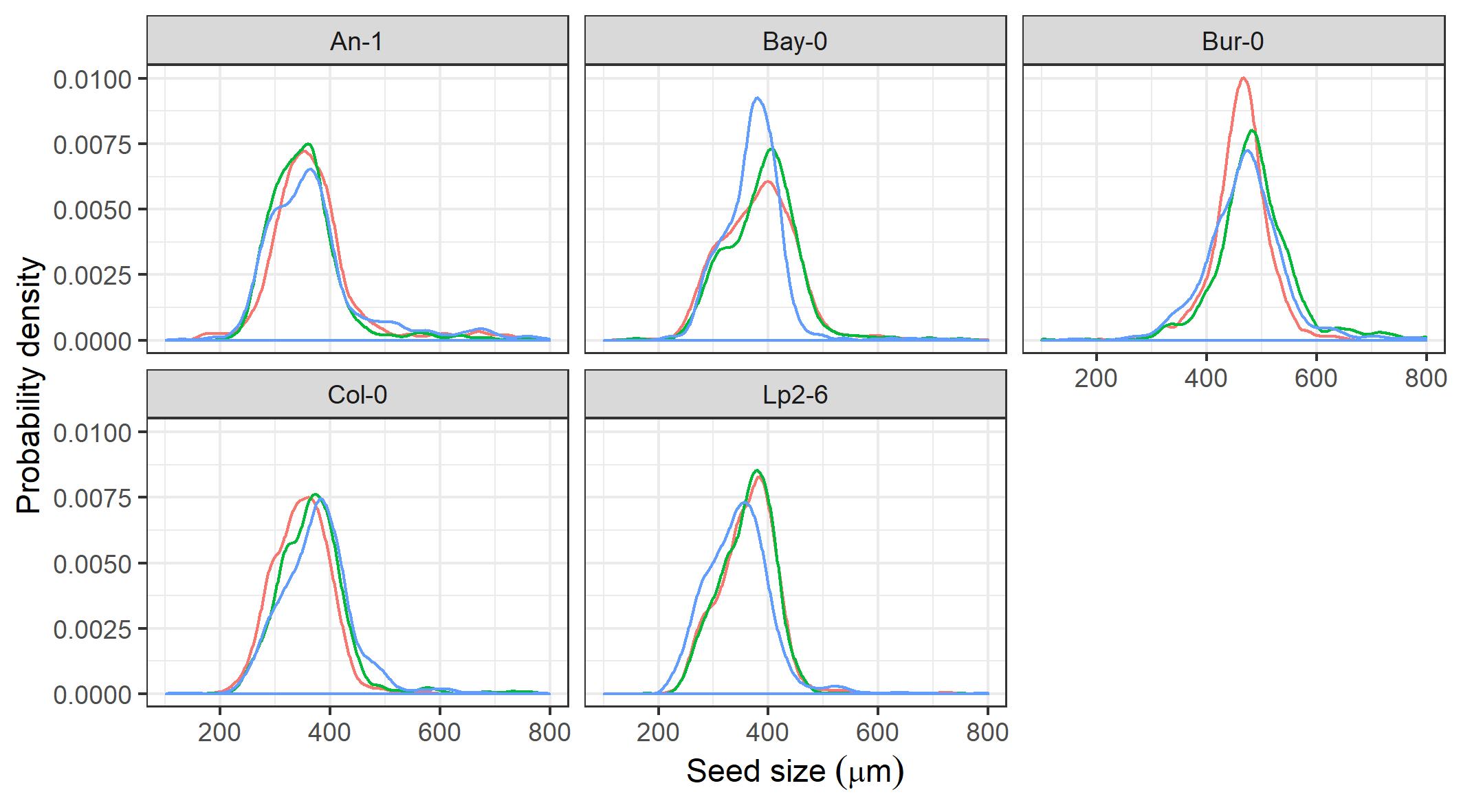


Figure S2: Distribution of seed sizes for the manually-cleaned seed samples of each of the five Arabidopsis accessions after removal of dust particles with clustering method. Each coloured line represents the distribution of sizes of all seeds derived from one individual plant of the three replicates used per accession.


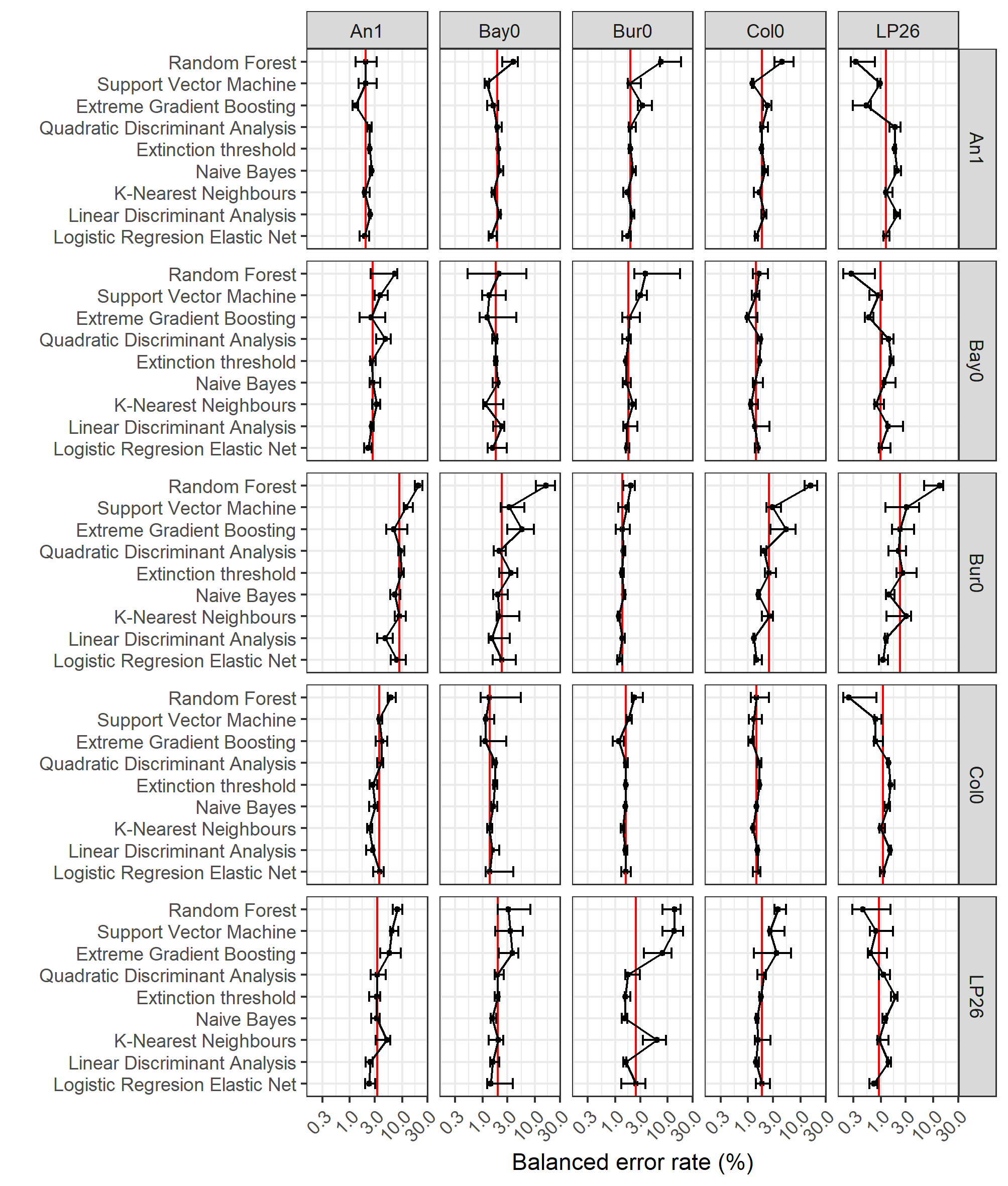


Figure S3: Matrix of plots of predictive median balanced error rate of each supervised classification algorithm within (IntraGenotype) and across accessions (InterGenotype). Columns and rows indicate the accessions on which the algorithm was trained and tested, respectively. The red lines indicate the median balanced error rate per plot whereas the error bars indicate the interquartile range (between percentiles 25% and 75%).


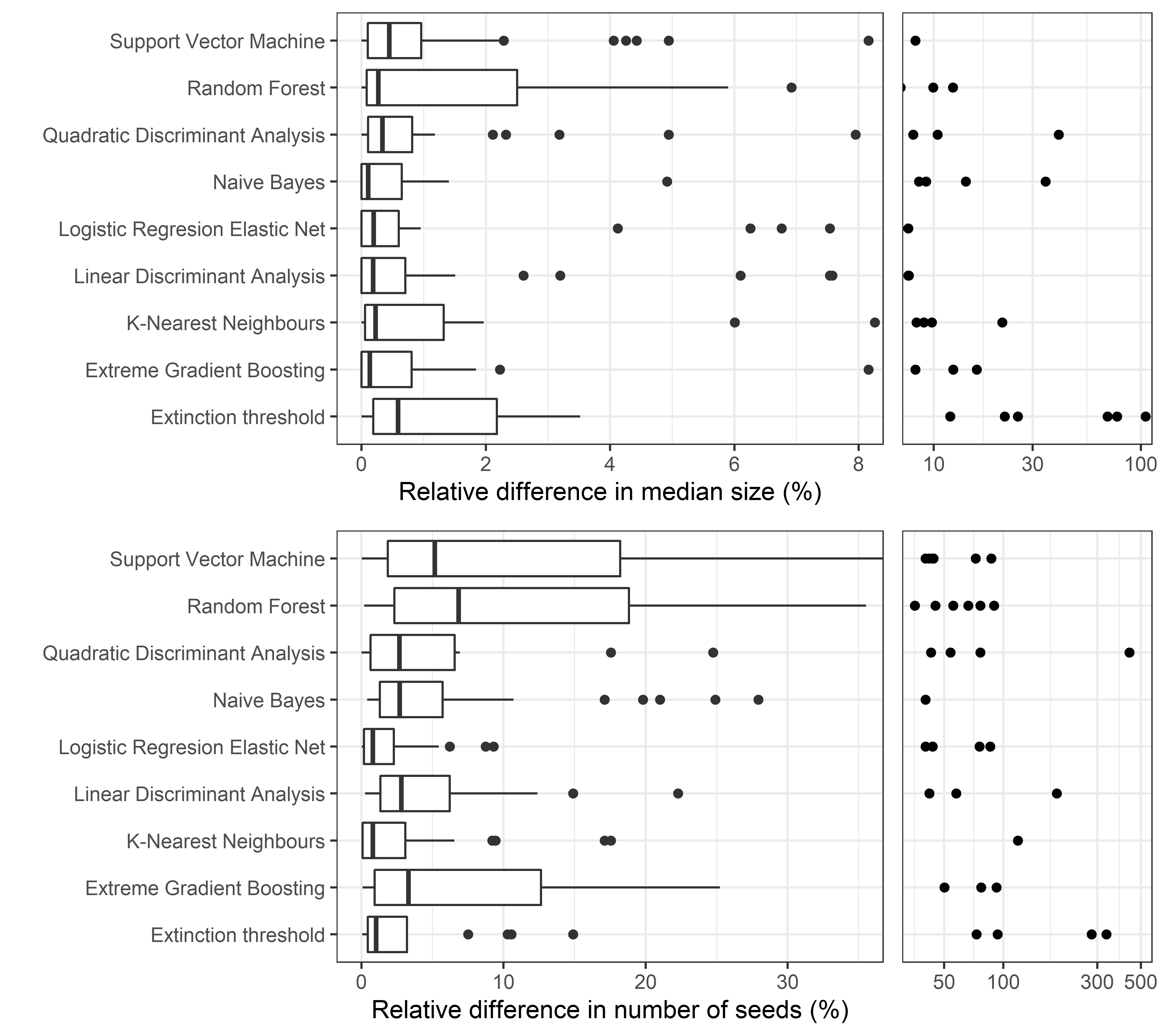


Figure S4: Boxplots of the relative differences in median seed size (upper panels) and number of seeds (lower panels) for each sample and algorithm with respect to the median values across all algorithms of the same quantities for each sample. Each panel is divided into two subpanels that represent different portions of the same data; note that the panels on the right side are on a log_10_ scale. The relative difference in variable *x* for algorithm *i* is calculated as ${|x_{i}-\mu_{x}|}/{\mu_{x}}$ where $\mu_{x}$ is the median value of *x* across all algorithms.

1. Hansen N, The CMA. Evolution strategy: a comparing review. In: Lozano JA, Larrañaga P, Inza I, Bengoetxea E, editors. Towards a new evolutionary computation: advances in the estimation of distribution algorithms. Berlin: Springer, Heidelberg; 2006. p. 75–102. [↑](#footnote-ref-1)
